# Drug Resistance in Non-B Subtype HIV-1: Impact of HIV-1 Reverse Transcriptase Inhibitors

**DOI:** 10.3390/v6093535

**Published:** 2014-09-24

**Authors:** Kamalendra Singh, Jacqueline A. Flores, Karen A. Kirby, Ujjwal Neogi, Anders Sonnerborg, Atsuko Hachiya, Kalyan Das, Eddy Arnold, Carole McArthur, Michael Parniak, Stefan G. Sarafianos

**Affiliations:** 1 Christopher Bond Life Sciences Center, University of Missouri, Columbia, MO 65211, USA; singhka@missouri.edu (K.S.); jaf468@mail.missouri.edu (J.A.F); kirbyk@missouri.edu (K.A.K.); 2 Department of Molecular Microbiology and Immunology, University of Missouri, Columbia, MO 65211, USA; 3 Division of Clinical Microbiology, Department of Laboratory Medicine, Karolinska Institute, Stockholm 141 86, Sweden; E-Mails: ujjwal.neogi@ki.se; (U.N.); Anders.Sonnerborg@ki.se (A.S.); 4 Clinical Research Center, Department of Infectious Diseases and Immunology, National Hospital Organization, Nagoya Medical Center, Nagoya 460-0001, Japan; E-Mail: hachiya@mail-nmc.jp; 5 Center for Advanced Biotechnology and Medicine, Rutgers University, Piscataway, NJ 08854, USA, E-Mails: kalyan@cabm.rutgers.edu (K.D.); arnold@cabm.rutgers.edu (E.A.); 6 Department of Chemistry and Chemical Biology, Rutgers University, Piscataway, NJ 08854, USA; 7 Department of Oral and Craniofacial Science , School of Dentistry, University of Missouri, Kansas City, MO 64108, USA; E-Mail: McArthurC@umkc.edu; 8 Department of Microbiology and Molecular Genetics, University of Pittsburgh School of Medicine, Pittsburgh, PA 15219, USA; E-Mail: map167@pitt.edu; 9 Department of Biochemistry, University of Missouri, Columbia, MO 65211, USA

**Keywords:** HIV-1 reverse transcriptase, HIV subtypes, nucleoside RT inhibitors, non-nucleoside RT inhibitors, translocation defective RT inhibitors, drug resistance

## Abstract

Human immunodeficiency virus (HIV) causes approximately 2.5 million new infections every year, and nearly 1.6 million patients succumb to HIV each year. Several factors, including cross-species transmission and error-prone replication have resulted in extraordinary genetic diversity of HIV groups. One of these groups, known as group M (main) contains nine subtypes (A-D, F-H and J-K) and causes ~95% of all HIV infections. Most reported data on susceptibility and resistance to anti-HIV therapies are from subtype B HIV infections, which are prevalent in developed countries but account for only ~12% of all global HIV infections, whereas non-B subtype HIV infections that account for ~88% of all HIV infections are prevalent primarily in low and middle-income countries. Although the treatments for subtype B infections are generally effective against non-B subtype infections, there are differences in response to therapies. Here, we review how polymorphisms, transmission efficiency of drug-resistant strains, and differences in genetic barrier for drug resistance can differentially alter the response to reverse transcriptase-targeting therapies in various subtypes.

## 1. Introduction

Approximately 35 million people are infected with different types and subtypes of HIV. Of the two major HIV types, HIV type 1 (HIV-1) causes ~95% of all infections, whereas HIV-2 (~5%) is mainly limited to West African countries (Senegal, Mali, Ghana, Guinea-Bissau, the Gambia, Niger, Cote d’Ivoire, and Liberia). HIV-1 has been divided into four groups: major (M), outlier (O), non‑M/non-O (N), and a new group P [1,2]. Group M accounts for more than 95% of all reported HIV‑1 infections [3].

Group M has been further divided into nine subtypes (also called clades): A-D, F-H, and J-K [2]. Of these, subtype C (HIV-1C) is the most prevalent worldwide and accounts for 50%–55% of all HIV infections [4]. It is mainly found in sub-Saharan Africa, Brazil, and India [4]. Subtype A (HIV-1A), which is approximately 20% of all HIV-1 infections, is primarily found in Eastern Europe, Northern Asia, and Western-Africa [4]. HIV-1 subtype B (HIV-1B) accounts for ~12% worldwide HIV-1 infections and is the main strain reported in developed countries (North America, Western Europe, Japan, and Australia). Other HIV-1 subtypes (D–K, or HIV-1D, HIV-1K, *etc.*) are rare (less than 6% of all infections). In addition to nine group M (HIV-M) subtypes, 66 circulating recombinant forms (CRFs) that evolve by recombination of two or more subtypes have been reported [5]. Although generally the geographic distribution of CRFs is not well defined, the CRF01_AE and CRF02_AG CRFs are predominately found in Southeast Asia and West Africa, respectively [6,7].

Most available drug-resistance data are derived from studies on HIV-1B, mainly because of the early availability of drugs and drug-resistance testing in developed countries. In contrast, similar data on non-B subtypes of HIV-1 (HIV-non-B) are limited, primarily due to historic delays in the availability of antiretroviral therapies (ART) in resource-limited countries. Increased access to ARTs, however, with significantly high non-adherence together with factors such as high rate of mutation, viral transmission and recombination in HIV-non-B infected patients have resulted in the emergence of drug-resistance data for other subtypes [8].

High genetic diversity of HIV-1 subtypes may influence the resistance pathways to currently used antiretrovirals (ARVs) [7]. The genetic differences can also impact the extent of cross-resistance to ARVs of the same class and lead to virologic failure, which may affect clinical outcome and immunological response [7,9]. Moreover, there is a considerable body of evidence that shows naturally occurring polymorphisms affect ARV response in subtype B virus [10,11,12,13,14,15,16,17]. A similar effect of polymorphisms can also be hypothesized for HIV-non-B viruses. In fact, recent reports have suggested that naturally occurring polymorphisms in HIV-non-B affect susceptibility to ARVs [18], although the impact of these polymorphisms on drug resistance is not well understood.

Current anti-HIV therapies, also known as highly active antiretroviral therapies (HAART), typically comprise combinations of nucleoside/nucleotide reverse transcriptase inhibitors (NRTIs), non-nucleoside reverse transcriptase inhibitors (NNRTIs), protease inhibitors (PIs), and integrase inhibitors (INSTIs). Generally, these ARVs are also effective against HIV-non-B viruses, even though they were originally designed and developed targeting HIV-1B virus. Nonetheless, susceptibility to ARVs can vary dramatically depending upon subtype due to inherent polymorphisms and cross-species transmission routes that may create differences in the drug resistance mechanisms of HIV-1 subtypes. Here, we summarize the status of HIV-non-B resistance to currently used ARTs in low- and middle-income countries, and present a comprehensive comparison with HIV-1B.

## 2. Resistance to Antiretroviral Therapies among Different HIV-1 Subtypes

**Figure 1:**
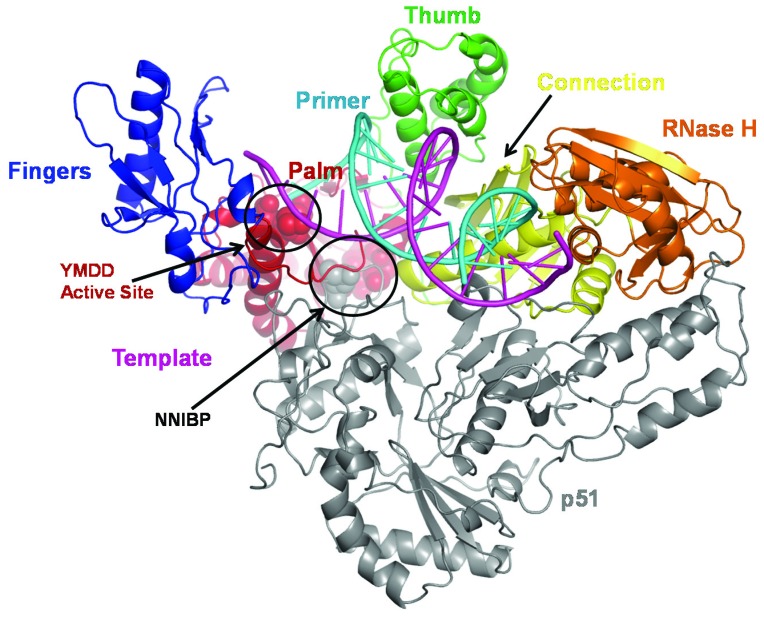
The crystal structure of HIV-1 RT bound to double stranded DNA. HIV-1 RT is a heterodimer of p66 and p51 subunits. Due to the resemblance of p66 to a half-open right hand, different subdomains in p66 have been named as the “palm” (red), fingers (blue), and thumb (green) [33]. The polymerase and RNase H (orange) activities are located in p66 subunit, and are linked by the connection subdomain (yellow). The p51 (dark gray) subunit is derived from proteolytic cleavage of the RNase H from p66 and has identical primary and secondary structure. However, the tertiary structure of p51 is markedly different than p66, leading to a non-functional arrangement of catalytic residues. The template/primer (magenta/cyan) binds in a DNA-binding cleft formed primarily by the p66 subunit. The active site (marked as YMDD) and the NNRTI binding pocket (NNIBP) are shown as space-filled atoms. This figure was generated from Protein Data Bank file 2HMI [20]. This, as well as Figure 4 and Figure 5, were generated using PyMOL program [36].

Six different classes of drugs have been approved by US Food and Drug Administration (FDA) [19] for HIV-1 treatment. The two drug classes: (i) NRTIs; and (ii) NNRTIs target the viral enzyme reverse transcriptase (RT). RT is a multi-functional enzyme. It carries out both RNA- and DNA-dependent DNA polymerase activities, as well as RNase H activity (Figure 1). All three activities are required for the synthesis of dsDNA from viral ssRNA genome. Structures of HIV-1B RT in various functional and inhibited states have been reported over the past 22 years. These include structures in (i) unliganded form; (ii) in complex with various template-primers (T/P); (iii) T/P and dNTP; (iv) T/P and NRTI‑triphosphates (TP); (v) NNRTIs; or (vi) nucleic acid and NNRTIs [20,21,22,23,24,25,26,27,28,29,30,31,32,33,34,35].

Structure(s) of HIV-non-B RTs have not been determined, although a structure of an unliganded HIV-2 RT has been reported [37]. However, due to the high sequence conservation between HIV-1B and HIV-non-B RTs, the overall folding of HIV-non-B RT is expected to be similar. RT is an asymmetric heterodimer comprising two structurally distinct subunits (p66 and p51). The p66 subunit contains the active sites for the polymerase and RNase H activities of the enzyme. The p51 subunit, which is derived from p66 by protease-mediated cleavage of the RNase H domain plays a structural role [26,32,33], although some mutations in p51 have been reported to affect catalytic activity as well [38,39].

**Figure 2:**
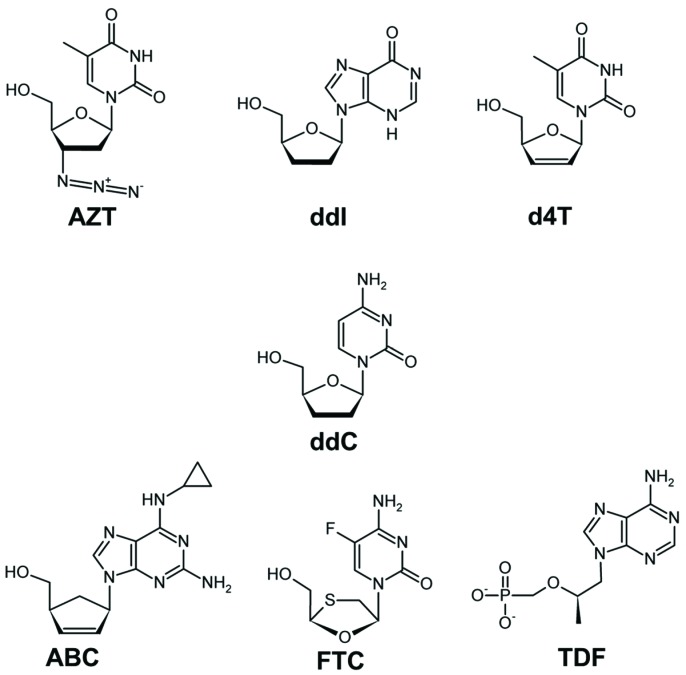
FDA approved nucleoside/nucleotide inhibitors (NRTIs/NtRTIs) of HIV-1B RT: All approved NRTIs/NtRTIs (AZT, azidothymidine; d4T, stavudine; FTC, emtricitabine; ddC, zalcitabine; ddI, didanosine; ABC, abacavir; TDF, tenofovir) lack a 3'-OH group. After incorporation of NRTIs/NtRTIs into DNA by HIV-1B RT, they act as chain terminators. FTC is 5-fluoro derivative of 3TC; the latter is not included in this figure. All chemical structures in this article were drawn by ChemSketch 3.5 [51].

The first anti-HIV drugs were nucleoside analogs lacking the 3'-OH group, which is required for DNA synthesis [40]. Currently, all approved NRTIs lack a 3'-OH (Figure 2), are phosphorylated in cells into dNTP analogs that bind at the dNTP-binding site [21,23,41,42], and act as chain terminators after their incorporation into viral DNA by RT [40,43,44].

**Figure 3:**
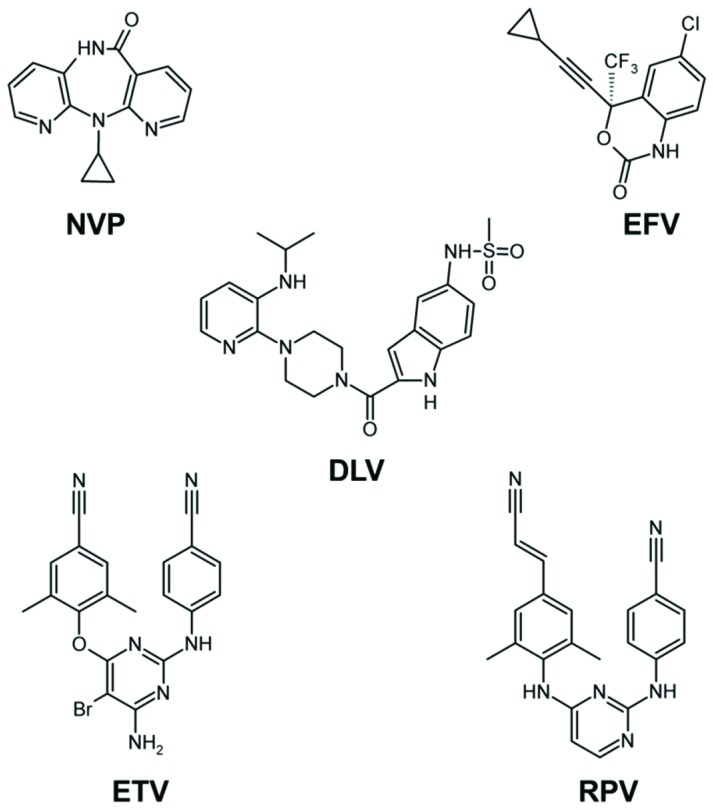
FDA-approved NNRTIs: nevirapine (NVP), efavirenz (EFV), delavirdine (DLV), etravirine (ETV), and rilpivirine (RPV). Currently, DLV is not recommended by the US Department of Health and Human Services. ETV, RPV, and sometimes EFV, are referred to as second-generation NNRTIs.

The second class of anti-HIV-1 RT drugs, (NNRTIs, Figure 3), bind in a hydrophobic pocket of HIV-1B RT at the base of the p66 thumb subdomain, ~10 Å away from the polymerase active site (Figure 1). NNRTI binding has multiple effects, including restriction of the p66 thumb subdomain’s flexibility, and repositioning of the nucleic acid [25,26,33,43,45,46].

### 2.1. Mechanism of NRTI Resistance in HIV-1B

**Figure 4:**
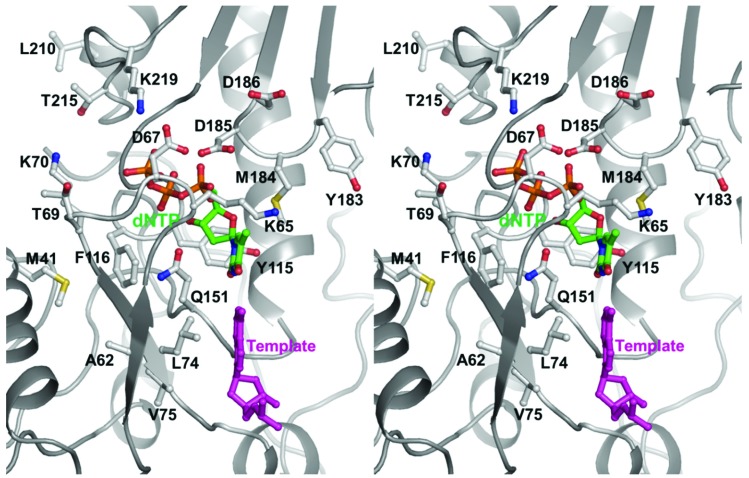
Stereoview of the polymerase active site of HIV-1B RT with residues involved in NRTI resistance. Amino acid residues, dNTP and template substrates are shown in ball-and-stick representation. The backbone of HIV-1 RT (PDB file 1RTD) [21] is shown in light gray ribbon representation. The amino acid side chains are colored by atom type (carbon, white; oxygen, red; nitrogen, blue; and sulfur, yellow). The dNTP is also colored by atom type but the carbon and phosphorus atoms are green and orange, respectively. The template nucleotide is colored magenta. For clarity, the position of F77 is not shown.

In HIV-1B, major mutations associated with NRTI resistance are M41L, A62V, D67N, 69 insertions (T69S plus the addition of SS, SA, or SG), K70R/E, K65R, L74V, V75I, F77L, Y115F, F116Y, Q151M, M184V/I, L210W, T215Y/F, and K219Q/E (Figure 4) [47]. Of these, M41L, A62V, 69ins, K70R, L210W, T215Y/F, and K219Q/E form the 69 insertion complex. Mutations A62V, V75I, F77L, F116Y, and Q151M combine to form the Q151 complex, and mutations M41L, D67N, K70R, L210W, T215Y/F and K219Q/E are known as TAMs (thymidine-associated mutations) or EEMs (excision-enhancing mutations) [47]. The 69 insertion complex is associated with resistance to all approved NRTIs when present with one or more TAMs at codons 41, 210, or 215 [47,48]. The Q151 complex mutations confer reduced susceptibility to all NRTIs except TDF (tenofovir) [48] while the TAMs enhance resistance to all approved NRTIs [49,50].

Mutations K65R, L74V, Y115F, and M184V are associated with resistance to abacavir (ABC), K65R and L74V are ddI-resistance mutations, K65R and M184V/I are resistant to FTC and 3TC, K65R and K70E are related to TDF resistance, and M41L, D67N, K70R, L210W, T215Y/F, and K219Q/E cause resistance to AZT [47].

Resistance to NRTIs is achieved by two distinct mechanisms in HIV-1B: (i) discrimination; and (ii) excision. In the discrimination mechanism, the mutations favor natural dNTP substrate over NRTI/NtRTI by decreasing binding affinity of NRTI-TP (K_d_), by reducing polymerization rate (*k*_pol_), or both [44,52,53,54,55,56]. The mutations known to cause resistance using the discrimination mechanism are K65R, K70E, L74V, V75I, Q151M complex, and M184V/I [52,53,55,57,58,59,60,61,62,63,64,65,66,67]. Biochemical data show that most of these mutations reduce the polymerase rate (*k*_pol_) and/or decrease the NRTI-TP binding affinity (K_d_) compared to the wild-type HIV-1B RT [40,43,52,53,55,57,58,59,60,61,62,63,64,65,66,67].

Using the excision mechanism, an EEM HIV-1B RT carries out ATP-mediated excision of NRTI from the 3'-end of the DNA primer chain and permits extension of the DNA [57,68,69]. This process is also referred to as “primer unblocking”. AZT and d4T resistance mutations M41L, D67N, K70R, L210W, T215Y/F, and K219Q/E are the most common examples of EEMs. These residues are located outside the dNTP-binding site [21,23,24,40,43,70,71,72] and do not affect the incorporation of nucleotide analogs [56,73,74,75,76,77]. Instead, they facilitate the ATP-dependent removal of the incorporated NRTI-MP that is catalyzed by the same active site as the polymerization reaction but in the reverse direction [69,70,78,79,80,81,82,83].

### 2.2. NRTI-Associated Mutations in Different HIV-1 Subtypes

A disparity in resistance mutations after NRTI treatment has been seen among HIV-1 subtypes. HIV-1C patients from Botswana who underwent AZT/ddI-containing HAART developed resistance predominantly through the 67N/70R/215Y pathway [84]. These mutations are different than AZT/ddI-associated resistance mutations (TAM/EEM) in HIV-1B. In HIV-1B, AZT/ddI resistance is acquired through either a TAM1 (M41L, L210W and T215Y) or a TAM2 (D67N, K70R and K219E/Q) pathway. A different set of mutations was selected in HIV-1C infected patients in India [85] under the same treatment protocol. While most of the NRTI-resistance mutations in the Indian patients were similar to those in HIV-1B (TAM1 and TAM2), they also harbored additional RT mutations A98G, E203D/K/V/N/A, H208Y, and H221Y [85]. Similarly, an entirely different resistance pattern was seen in HIV-1C infected patients from Malawi and South Africa who received first-line treatment with d4T/3TC/NVP [86,87]. Not surprisingly, the most prevalent NRTI-associated mutation in these patients was M184V (81%), which is associated with 3TC treatment. Interestingly, 23% of the patients acquired the K70E or K65R mutations that are associated with TDF resistance even though the patients were not exposed to TDF [86]. Results from another study in Botswana also showed high incidences of the K65R mutation (30%) in HIV-1C patients who received combinations of either d4T/ddI/NVP or d4T/ddI/EFV [88]. The K65R mutation was detected in 7% and 15% of patients in South Africa failing first- and second-line regimens, respectively [89,90,91]. The nucleoside backbone in these treatments included d4T/3TC or ddI/AZT [90,91]. The K65R mutation in HIV-1C was also seen in Ethiopian and Indian patients (10%–12%) who failed d4T/3TC/NVP therapy [92,93]. These results suggest that the K65R substitution may emerge at a higher frequency among individuals infected with HIV-1C viruses who are receive d4T/3TC-based first-line therapy, and the introduction of TDF-based regimens may show limited success [9].

The K65R mutation has been shown to be selected faster in HIV-1C than in HIV-1B when tenofovir (TDF) is used as one of the nucleoside drugs [7]. On the contrary, K65R appears to be less frequent in HIV-1A than other subtypes [94]. Some subtype-dependent differences in K65R or TAM mutations can be attributed to treatment regimens, disease stage and access to viral load testing in different developing countries [94]. The faster acquisition of K65R in HIV-1C has also been linked to the nature of the HIV-1CRNA template [95,96,97].

### 2.3. NNRTI-Associated Mutations in Different HIV-1 Subtypes

**Figure 5:**
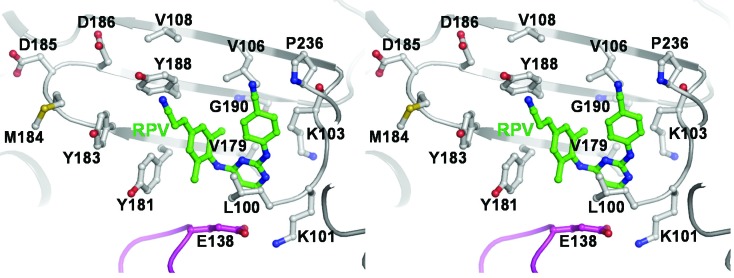
Amino acids involved in HIV-1B NNRTI resistance: Stereoview of HIV-1B RT with RPV bound at the NNIBP and NNRTI-resistance residues and RPV shown in ball-and-stick representation. The RT backbone (PDB file 2ZD1) [30] is shown in ribbon representation (p66 in light gray and p51 in magenta). Amino acid side chains are colored with carbon in white, oxygen in red, nitrogen in blue, and sulfur in yellow. RPV carbons are shown in green and nitrogens in blue. RPV resistance residue E138 [103,104,119,120,121] is shown in magenta. Residues 227, 229, and 234 are omitted for clarity.

EFV and NVP are two NNRTIs that have been widely used in both developed and resource-limited countries. However, both NNRTIs have a low genetic barrier [98]. A single mutation in the NNRTI binding pocket is sufficient to cause clinical failure [98]. Moreover, a high level of cross-resistance between NVP and EFV [99] has limited available therapeutic options. ETV and RPV are second generation NNRTIs that are effective against common NVP and/or EFV resistant HIV-1 viruses [100,101,102]. NNRTIs bind to a hydrophobic pocket (non-nucleoside inhibitor binding pocket or NNIBP) formed by residues 95, 100, 101, 103, 106, 108, 179, 181, 188, 190, 227, 229, 234, 236, and 318 from the p66 subunit and 138 from the p51 subunit (Figure 5) [27,30,45]. NNRTI resistance can be achieved by two different mechanisms: (i) affecting the entry/release (*k_on_*/*k_off_*) of NNRTI to and from the NNIBP (e.g., K103N and E138K mutations) [38,103,104,105,106,107]; and (ii) altering the NNIBP geometry, thus directly affecting interactions with the inhibitor (e.g., Y181C and Y188L) [27,29,72,108,109]. 

The NNRTIs are used by themselves and in combination with two NRTIs. Similar to diverse mutation pattern in response to NRTI treatment, different patterns of NNRTI-resistance mutations have been reported for different HIV subtypes. Single-dose NVP treatment, often used for prevention of mother-to-child transmission (PMTCT) in countries with limited resources, has shown a wide range of subtype-dependent resistance patterns. Resistance to NVP monotherapy in HIV-1C, HIV-1D, HIV1-A and HIV-1 CRF02_AG infections has been reported to be 69%, 36%, 19%, and 21%, respectively [110]. Similarly, a meta-analysis in South African HIV-1C patients treated for PMTCT showed that 35.7% of women and 52.6% of children carried virus with NVP-resistance mutations [111]. Results from a Ugandan cohort showed that HIV-1C was more NVP resistant than HIV-1D and HIV-1A [112]. The K103N mutation most frequently appeared in mothers, while the Y181C mutation was predominant in children [113]. These results clearly demonstrate that the response to NVP monotherapy (as for PMTCT) significantly varies among HIV subtypes.

During *in vitro* serial passages of HIV-1C in NVP or EFV, the virus developed the V106M mutation, in addition to the V106A mutation, which is commonly selected in HIV-1B [114]. This difference has been attributed to a nucleotide polymorphism at position 106 of RT [18]. The clinical importance of V106M in HIV-non-B subtypes has been confirmed in many studies, which show that V106M emerges frequently in patients infected with HIV-1C and HIV-1 CRF01_AE viruses during therapy with NVP or EFV [80,84,85,115,116,117,118]. It should be noted that, similar to residue 106, additional silent drug-resistant RT mutations at residues 65, 138, and 161 were reported in Ethiopian isolates and subtype C reference strains [114].

Data from the DUET-1 and DUET-2 phase III clinical trials revealed a total of 17 ETV resistance-associated mutations (RAMs) (V90I, A98G, L100I, K101E/H/P, V106I, E138A, V179D/F/T, Y181C/I/V, G190A/S, and M230L) [100,101]. Three or more of these mutations were associated with decreased virological response to ETV [122]. V90I and E138A were the most prevalent mutations in HIV-1 CRF02_AG and HIV-1A1, while V106I was most prevalent in HIV-1C and HIV-1D. Biochemical data showed that E138A in combination with V179E and Y181C had increased resistance to ETV by five-fold and 11-fold, respectively [122]; however, none of the dual mutation samples were found to be ETV resistant [122].

*In vitro* serial passages of HIV-1B, HIV-1C, and HIV-1 CRF02_AG in ETV showed distinct patterns of resistance mutations [123]. HIV-1C acquired the V90I, V106M, E138K, V179D/E, Y181C, G190E/G, M230L, and K238N mutations. These mutations also emerged in HIV-1 CRF02_AG. However, in addition to the above mutations (except for M230L), ETV also selected K101Q, V189I, and H221Y in HIV-1B. Interestingly, the K103N/Q mutations were selected in HIV-1B and HIV-1 CRF02_AG, but not in HIV-1C.

In low- and middle- income countries (LMICs) where phenotyping and genotyping is not widely available and where non-B strains dominate, the patients’ failure to a treatment regimen is always monitored either by immunological criteria (CD4^+^ T-cell counts) or manifestation of clinical symptoms. In such cases, extensive cross-resistance is observed, even when NVP and EFV are replaced with the second generation NNRTI etravirine (ETV). A study from India reported that in a failing regimen including NVP or EFV, 40% of the HIV-1C infected patients had at least partial resistance to ETV [124]. Similar observations were reported in another Indian study where the HIV-1C-infected patients were treated with NVP/EFV for one to eight years and 45% were resistant to ETV [93].

A group of 17 mutations, L100I, K101E/P, E138A/G/K/Q/R, V179L, Y181C/I/V, Y188L, H221Y, F227C, and M230I/L, have been defined as RPV RAMs [47,125,126,127,128]. The prevalence of these mutations among RPV treatment-naïve (but treated with NRTI+PI or NRTI+NNRTI regimens) patients infected with HIV-1B and HIV-non-B has been reported [125]. Nearly 5% of the patients harbored primary RPV mutations. E138A was the most prevalent mutation (3.2%) followed by E138K, H221Y, E138G, Y181C, and Y188L in HIV-1C. The distribution of RPV RAMs was significantly different between HIV-1B (2%) and HIV-non-B (5%) subtype patients [125]. This difference can be attributed to E138A, which is a known polymorphism mutation (6%–8%) in HIV-1C virus [129].

Despite the structural similarity between ETV and RPV, both being DAPY (diarylpyrimidine) derivatives, different sets of resistance mutations have been reported for the same HIV-non-B (HIV-CRF01_AE) infection. The mutations Y181C, G190A, and K103N emerged in patients who failed ETV therapy, whereas mutations K101P, Y181I, and Y181V were predominantly seen in patients who failed RPV [130]. Notably, the patients who failed RPV therapy were resistant to ETV even though ETV RAMs were not found among these patients [125,130]. Many other NNRTI-resistance mutations that vary from subtype to subtype have been reported. A list of these mutations has been is given in Table 1. In some subtypes, resistance to given NNRTI(s) has been attributed to a high degree of polymorphism. For example, A98S mutation, which is resistant to all NNRTIs in HIV-1B and HIV-1C (Table 1) may confer resistance to NNRTIs in HIV-1G due to high polymorphism in this subtype [131].

**Table 1:** NNRTI-resistance mutations in response to NNRTI treatment among different HIV subtypes (modified from Santoro and Perno, [132]).

Mutation	Subtype	NNRTIs	Comment	Reference
K103N	B, C, F, CRF02_AE	EFV, DLV, NVP	K103 appears with low frequency in C compared to B, F and CRF02_AE	[133,134,135,136]
V106M	B, C and CRF01_AE	EFV, NVP	V106M emerges frequently in C due to low genetic barrier compared to B and CRF01_AE	[99,114,118]
E138K	B, C, CRF02_AG	ETR	First emerges in B, C and CRF02_AG. Preferential selection in B is Y181C	[80,123]
G190A	C	All NNRTIs	This mutation in C may be due to high G190A/S polymorphism in C	[118]
Y181C	A, B	ETR	Preferential selection under drug pressure on A and B subtypes	[80]
Y181C and Y188L	C	EFV, DLV, NPV	High frequency in C	[133,134,135,136]
N348I	C	ETR	High frequency in C at ETR failure	[137,138]
N348I	B	NVP	High frequency in B at NVP failure	[11,12]

### 2.4. Mutations that Impact both Classes of RTIs (Connection Subdomain Mutations)

Until recently, all HIV-1 drug-resistance testing kits interrogated ~250 *N*-terminal amino acids of HIV-1 RT. These testing kits were based on the assumption that all currently approved RTIs target the polymerase domain of RT, and mutations in the domain confer drug resistance. One group of researchers from Japan, in collaboration with us [11] and another group from Australia [12], independently showed that the connection subdomain mutation N348I causes resistance to both classes of drugs (NRTIs and NNRTIs) in HIV-1B and HIV-1D patients. Biochemical data showed that N348I, by itself or in combination with TAMs, reduces the rate of RNA template degradation by RT [12,139]. We used transient-state kinetics to decipher the contribution of N348I in the polymerase function of RT and resistance to NVP [38]. We demonstrated that the N348I mutation increases the processivity of RT and enhances NVP dissociation from the NNIBP [38]. A molecular dynamics modeling study proposes that N348I contributes to NVP resistance through a long-range allosteric communication network between the connection and NNIBP [140]. More recently, Brehm *et al.* [138] reported the presence of the N348I mutation in HIV-1C patients who failed d4T/3TC/NVP or d4T/3TC/EFV therapy. A group of drug-resistance mutations in the connection subdomain of RT (A376S, A400T, Q334D, G335D, N348I, and A371V) in a Brazilian cohort (HIV-1C) was recently identified using single genome sequencing methodology [141]. The connection subdomain mutations identified in this study were similar to those previously reported in subtype B. However, the data showed that these mutations enhanced resistance to ETV by six- to 11-fold in the presence of L100I/K103N/Y181C (NNRTI) and TAM-associated mutations [141]. A detailed kinetic characterization of connection subdomain mutations in HIV-non-B needs to be performed before one can establish whether their mechanism of inhibition and resistance is similar to that in HIV-1B.

## 3. Transmitted Drug Resistance (TDR) in HIV-Non-B

Transmitted drug resistance (TDR) is defined as resistance to one or more ARVs in individuals with no previous drug exposure, and it is presumed to be the result of the direct transmission of resistant strains from treated individuals [43]. TDR can be complicated by the presence of drug resistance mutations in a given subtype that may exist as polymorphisms in some other subtype. Both TDR and polymorphisms can lead to ART failure in treatment-naïve patients and limit the efficacy of drug regimens. In regions of the world where HAART has been used for several years, TDR can be found in up to 25% of HIV infected individuals [128,142,143,144]. The most commonly reported NRTI TDR mutations in HIV-1B are M184V and M184I. Other TDR mutations are M41L, K219E/N/Q/R, and T215F/Y and 215 revertant mutations (T215D/C/E/S/I/V) [145,146]. Transmission of M184V has also been reported in HIV-non-B [143] cases with high-level resistance to 3TC and FTC. The most common HIV-non-B TDR mutations are K103N, Y181C/I, and Y188C/H/L [143,144,147,148]. 

## 4. Polymorphisms in HIV-Non-B 

A polymorphism is a nucleotide variation from the consensus sequence within a subtype, arbitrarily defined as a position-specific difference occurring in >1% of sequences [147]. Biochemical and virological data have demonstrated that polymorphisms can affect the magnitude of resistance conferred by known resistance mutations [10,11,15,148,149], not only in different HIV-1 subtypes but also in different HIV types. For example, HIV-2 and group O viruses have high-level innate resistance to NNRTIs because of drug-resistance polymorphisms [150,151]. Of the first 240 amino acid residues of HIV-1 RT, polymorphisms at 41 positions in HIV-1A (17%), 45 in HIV-1B (19%), 56 in HIV-1C (23%), 43 in HIV-1D (18%), 35 in HIV-1F (15%), 32 in HIV-1G (13%), 46 in HIV-1 CRF01_AE (19%), and 56 in HIV-1 CRF021_AG (23%) have been reported based on testing of drug-naïve HIV-infected individuals [131,147]. Subtype-specific polymorphisms at residue positions that are related to NRTI and NNRTI drug resistance are shown in Table 2.

**Table 2:** Polymorphism at drug resistance positions in RT from untreated patients (modified from Kantor and Katzenstein, [147]).

Subtype	Subtype Reference Sequences	RT Sequence PM Position, NRTI RAMs	RT Sequence PM Position, NNRTI RAMs
A	U455	A62V, V118I	E138A, V179I/T, K238R
B	HXB2	V118I	A98S, V179I/D/E
C	C2220	V118I	A98S, E138A, V179I
D	NDK	V118I (comparable to B)	V179I
F	93BR020	V41I, M184V	E138A/G, V179I/D
G	SE6165		A98S, V179E
CRF01_AE	CM240	V41I, A62V	V75L, V106I, V179I/D
CRF02_AG	IbNG	V41I, V118I	V106I, V179I

It is now well established that connection subdomain mutations are associated with low-level resistance to NNRTIs [11,12,152]. Several connection subdomain mutations are polymorphic in HIV-non-B [153,154,155]. Polymorphisms G335D and A371V in HIV-1 CRF01_AE showed significantly reduced susceptibility to NRTIs and NNRTIs [154]. In a Brazilian HIV-1C cohort, polymorphisms G335D, A376S, and A400T/S have been reported [153]. However, more data from different HIV-non-B subtypes are required to establish the effect of polymorphisms on drug susceptibility and drug resistance. 

## 5. Therapeutic Response in HIV-Non-B subtypes in the Presence of Drug Resistance Mutations

Several clinical studies have provided evidence of viral re-suppression despite the presence of major drug-resistance mutations (DRMs) in HIV-1C. A South African study showed that despite the presence of key NNRTI mutations like K103N, V106M, or G190A, a portion of patients achieved viral re-suppression [156]. This was further supported by a report from another South African cohort where, in nine of 12 viremic patients, HIV-1C was suppressed even in the presence of major DRMs, such as K103N or M184V [157]. A recent study from India, where HIV-1C is the prevalent subtype, reported that three patients had controlled viremia in the presence of multiple major NRTI and NNRTI resistance-associated mutations (M184V+G190A; M184V+K103N; M184V+Y181C) without change in therapy; the patients maintained >95% adherence both before and after the viral rebound as well as CD4^+^ T-cell counts in the normal range [158]. Collectively, these studies support the idea that further work is needed to understand how DRMs phenotypically affect viral fitness, drug susceptibility, and patients’ therapeutic responses.

## 6. Future Perspectives: New Class of NRTIs

**Figure 6:**
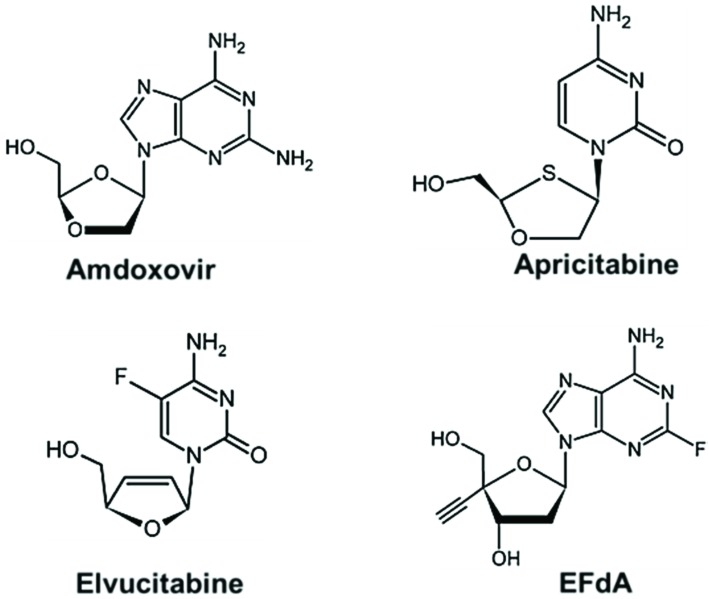
NRTIs in clinical trials or pre-clinical development. Amdoxovir, apricitabine, and elvucitabine are being developed by RFS Pharma, Avexa Pharmaceuticals, and Achillion Pharmaceuticals, respectively. EFdA has been licensed by Merck from Yamasa Corporation. With the exception of EFdA, these compounds lack a 3'-OH and function as chain terminators. EFdA contains a 3'-OH and blocks viral replication by multiple mechanisms, including by acting as a translocation-deficient reverse transcriptase inhibitor [103,163,164,168].

The combination of NRTIs currently in use can limit treatment efficacy in many ways [159]. For example, AZT and d4T share the same excision pathway and therefore combining the two drugs can produce suboptimal results. Similarly, drug-drug interactions such as those reported between abacavir (ABC) and other protease inhibitors (atazanavir/ritonavir or lopinavir/ritonavir) may also impact the outcome of some combination therapies [160]. The low genetic barrier of NNRTIs, higher polymorphism in NNIBP of HIV-non-B subtypes, and extensive cross-resistance to second generation NNRTIs after failure to first generation NNRTIs may present challenges for use of NNRTI-containing combinations in some cases. NRTIs that act by novel inhibition mechanisms, which can be used synergistically with current drugs to improve resistance profiles, are continuously pursued. The chemical structure of some new NRTIs, which are either in pre-clinical development or at various stages of clinical trials, are shown in Figure 6. Amdoxovir is a prodrug of β-d-dioxolane guanosine (DXG), currently evaluated in phase II clinical trials. Apricitabine (ATC) is in phase IIb clinical trials and it has been shown to be effective against HIV-1 viruses containing M184V, L74V, and TAMs [161].

Several 4'-substituted NRTIs have been reported to be potent antivirals [103,162,163]. Among them, 4'-ethynyl-2-fluoro-2'-deoxyadenosine (EFdA) (Figure 6) is the most potent that can inhibit RT as a translocation-defective RT inhibitor or as a delayed chain terminator, depending on the sequence of the template/primer substrate [164,165]. These compounds contain an ethynyl group modification at the 4' position of the sugar moiety that appears to make extensive interactions in a hydrophobic pocket of the RT polymerase active site and block the translocation of RT after their incorporation into the nascent DNA chain [163]. EFdA inhibits efficiently essentially all drug-resistant strains [166], and displays low mitochondrial toxicity due to the fact that it is a poor substrate for DNA polymerase γ [164,167]. The effect of these new NRTIs on HIV-non-B subtypes is yet to be determined.

## 7. Conclusions

In summary, the available biochemical, virological and clinical data on HIV-1B and HIV-non-B indicate that the genetic diversity among HIV-1 subtypes can impact the outcome of ART. As HAART becomes more accessible to more patients in resource-limited settings, a better picture of susceptibility and resistance is expected to emerge. These results may influence (i) the formulation of currently approved drugs and drug combinations to improve efficacy and adherence in HIV-non-B infected patients and (ii) the development of more potent and broad-spectrum NRTIs and NNRTIs.
